# Applications of Artificial Intelligence in Food Industry

**DOI:** 10.3390/foods14071241

**Published:** 2025-04-01

**Authors:** Heera Jayan, Weiqing Min, Zhiming Guo

**Affiliations:** 1 School of Food and Biological Engineering, Jiangsu University, Zhenjiang 212013, China; heerajayan93@outlook.com; 2 China Light Industry Key Laboratory of Food Intelligent Detection & Processing, Jiangsu University, Zhenjiang 212013, China; 3 Key Laboratory of Intelligent Information Processing, Institute of Computing Technology, Chinese Academy of Sciences, Beijing 100086, China

With breakthroughs in artificial intelligence (AI) brought by the fourth industrial revolution, intelligent applications are providing innovative solutions across food industry [1,2]. The powerful capabilities of AI in perceiving, reasoning, and decision-making are supporting the transformation and upgrading of food technologies, enabling automation, digitization and intelligence throughout the sector [3,4,5]. Novel AI based applications are improving every aspect of the food industry from production, processing and packaging to transportation, storage, consumption and waste management, thus benefiting all stakeholders in the food industry [6,7]. When integrated with complimentary techniques such as the Internet of Things (IoT) and Big Data, AI facilitates advancement such as development of new product, food process simulation and optimization, and enhanced monitoring of food quality and safety (Figure 1) [8,9,10].

Development of cutting-edge methodologies for assessing food quality and nutritional content remains a significant focus for researchers, particularly in fruits and vegetables [11,12]. Ropelewska & Szwejda-Grzybowska [13,14] demonstrated strong correlations between image texture parameters and chemical properties of red and yellow pepper, highlighting the potential of multispectral imaging for broader applications in food analysis. Another notable contribution is to integrate deep learning to evaluate the chemical composition and nutritional content of food product [15,16,17]. Additionally, the combination of deep learning and near-infrared spectroscopy has proven highly effective for determining soluble solid content (SSC) in apples [18,19]. The use of ant colony optimization algorithm achieved superior results for SSC prediction while also excelling in maturity classification based on apple color characteristics. Furthermore, the integrative technique was applied to assess the quality of dried kiwifruit, emphasizing the importance of visual attributes in predicting moisture content and optimising drying processes [20]. These studies display the significant potential of integrating deep learning with advanced imaging and spectroscopic technique for evaluating food quality and nutritional content, paving the way for more efficient and accurate food analysis [21,22].

The classification of fruits and vegetables based on the quality, authenticity maturity is also vital for informed consumer choices and efficient food processing. Noutfia & Ropelewska [23] highlighted the application of image analysis to classify date fruits into categories based on the drying processes, achieving over 94% accuracy for fresh, convective-dried, and infrared-dried samples across tested cultivars. Despite the availability of various methods for classification based on quality parameters, automating the entire process remains crucial for achieving real-time, on-line classification at an industrial scale [24,25]. Granados-Vega et al. [26] addressed this need by developing a prototype for Classification of Persian lemon artificial using neural network (ANN) trained on digital images. The ANN successfully classified 96.60% of samples, and simulations confirmed the feasibility of implementing this prototype in practical settings. Zhu et al. [27] introduced an on-line classification system for maize seeds based on the vitality, utilizing near-infrared spectroscopy data with machine learning models. Their approach achieved a classification accuracy exceeding 91%, highlighting the potential of NIR-based devices for real-time classification of agricultural products. These studies represent a significant leap forward in the automation of fruit and vegetable classification by harnessing the power of advanced machine learning and computer vision techniques [28,29].

Beyond component analysis and classification, machine learning and AI excel in optimizing food industry processes, enabling increased efficiency, better yields, and cost reductions [30,31]. Pao-la-or et al. [32] employed Artificial Neural Fuzzy Intelligent System (ANFIS) to determine the optimal extraction condition for supercritical fluid extraction of oil from pomegranate seeds. In a related study, Pao-la-or et al. [33] utilized deep learning to optimize the drying process of fingerroot extract. Their model successfully preserved bioactive compounds, enhancing the potential applications. This work demonstrated the effectiveness of AI in refining extraction process. On the other hand, AI based systems can track the movement of food products through the supply chain, enabling rapid identification and recall of contaminated products, ensuring food safety [34]. Deep learning techniques have been proven to provide information regarding the risk associated with food products and their potential recalls by relying on historical data [35].

**Figure 1:**
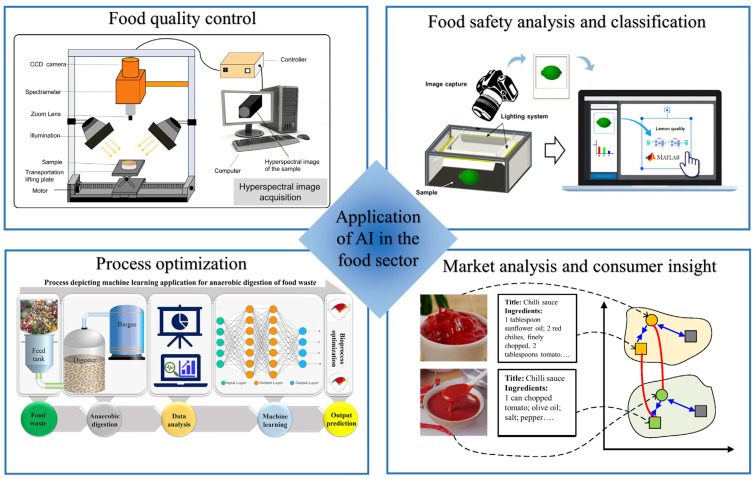
Application of AI in various field of food industry [26,36,37,38].

AI has the unique ability to process vast amounts of unstructured data from social media platforms, online reviews, and surveys, which will help in understanding emerging preferences and market gaps [39,40,41]. For example, Tao et al. [42] developed a tool to mine social media data for detecting foodborne illness trends, which could also provide insights into food safety and quality concerns of consumers. Similarly, Zuo et al. [38] proposed a Multi-Modal Alignment Method to retrieve recipes based on food images, highlighting the integration of cross-modal data to understand consumer preferences better and support tailored product development. Additionally, Chen et al. [43] introduced a health-aware food recommendation system leveraging a collaborative recipe knowledge graph and multi-task learning to provide personalized recommendations that balance dietary preferences with health requirements. This approach ensures consumers receive options tailored to both taste and health needs [44,45,46]. AI revolutionizes the food industry by empowering businesses to identify emerging trends, optimize formulations, and create personalized food recommendations [47]. This accelerates innovation and provides a strategic advantage in the rapidly evolving market.

Although AI based tools have been applied across various areas of food industry throughout the food supply chain, a significant gap remains between the development of AI technologies and their practical application [48,49,50]. This is especially evident in the fields of crop and agricultural sciences, where the integration of AI remains limited. Zhang et al. [51] recognized the potential of a multimodal technique that integrates AI with common non-destructive methods, demonstrating enhanced comprehensiveness and accuracy in food safety analyses. For example, Guo, et al. [52] combined SERS sensors with intelligent algorithms to achieve highly sensitive and in-situ detection of pesticides on apple. Further, extraction of useful information from temporal data is an extremely relevant research area, presenting significant opportunities for further exploitation in the food domain. Such advancement would allow AI to address complex challenges in the food industry, paving the way for effective and innovative solutions [53,54,55].

The future of AI applications in the food industry expected involve enhanced integration of technologies such as IoT, blockchain, and machine learning [56,57,58]. This integrative approach potentially enhance supply chain transparency, improve food safety, and optimize production processes [59]. In the future, AI is estimated to play a pivotal role in advancing sustainability by optimizing resource utilization, minimizing waste, and promoting environmentally sustainable practices across the entire food supply chain [60,61,62]. Further AI will remain as an integral part of developing personalized nutrition strategies and the development of products catered to meet the individual dietary requirement and choices [63,64,65]. For instance, deep generative networks can be employed to model user-specific information to generate accurate recommendations according to the guidelines. Additionally, AI-powered chatbots can engage in conversation regarding customer dietary preference and provide immediate suggestions and feedbacks [66,67].

## Data Availability

No new data were created or analyzed in this study. Data sharing is not applicable to this article.
